# Neural Dynamics in Primate Cortex during Exposure to Subanesthetic Concentrations of Nitrous Oxide

**DOI:** 10.1523/ENEURO.0479-20.2021

**Published:** 2021-07-14

**Authors:** Matthew S. Willsey, Chrono S. Nu, Samuel R. Nason, Karen E. Schroeder, Brianna C. Hutchison, Elissa J. Welle, Parag G. Patil, George A. Mashour, Cynthia A. Chestek

**Affiliations:** 1 Department of Neurosurgery, University of Michigan, Ann Arbor, Michigan 48109; 2 Department of Biomedical Engineering, University of Michigan, Ann Arbor, Michigan 48109; 3 Department of Neuroscience, Columbia University, New York, New York 10027; 4 Neuroscience Graduate Program, University of Michigan Medical School, Ann Arbor, Michigan 48109; 5 Department of Anesthesiology, University of Michigan, Ann Arbor, Michigan 48109; 6 Center for Consciousness Studies, University of Michigan, Ann Arbor, Michigan 48109; 7 Department of Electrical Engineering and Computer Science, University of Michigan, Ann Arbor, Michigan 48109; 8 Robotics Graduate Program, University of Michigan, Ann Arbor, Michigan 48109

**Keywords:** anesthesia, consciousness, nitrous oxide, NMDA antagonist, sensorimotor

## Abstract

Nitrous oxide (N_2_O) is a hypnotic gas with antidepressant and psychedelic properties at subanesthetic concentrations. Despite long-standing clinical use, there is insufficient understanding of its effect on neural dynamics and cortical processing, which is important for mechanistic understanding of its therapeutic effects. We administered subanesthetic (70%), inhaled N_2_O and studied the dynamic changes of spiking rate, spectral content, and somatosensory information representation in primary motor cortex (M1) in two male rhesus macaques implanted with Utah microelectrode arrays in the hand area of M1. The average sorted multiunit spiking rate in M1 increased from 8.1 ± 0.99 to 10.6 ± 1.3 Hz in Monkey W (*p *<* *0.001) and from 5.6 ± 0.87 to 7.0 ± 1.1 Hz in Monkey N (*p *=* *0.003). Power spectral densities increased in beta- and gamma-band power. To evaluate somatosensory content in M1 as a surrogate of information transfer, fingers were lightly brushed and classified using a naive Bayes classifier. In both monkeys, the proportion of correctly classified fingers dropped from 0.50 ± 0.06 before N_2_O inhalation to 0.34 ± 0.03 during N_2_O inhalation (*p *=* *0.018), although some fingers continued to be correctly classified (*p *=* *0.005). The decrease in correct classifications corresponded to decreased modulation depth for the population (*p *=* *0.005) and fewer modulated units (*p *=* *0.046). However, the increased single-unit firing rate was not correlated with its modulation depth (*R*^2^ < 0.001, *p *=* *0.93). These data suggest that N_2_O degrades information transfer, although no clear relationship was found between neuronal tuning and N_2_O-induced changes in firing rate.

## Significance Statement

There are few intracortical studies characterizing the influence of nitrous oxide (N_2_O) on neuronal behavior in the primate brain. Herein we demonstrate increased spiking rate in primary motor cortex (M1) as well as increased beta/gamma power during the administration of subanesthetic N_2_O. In a previously validated model of primary somatosensory to M1 information transfer, we also show a degradation of somatosensory representation. The degraded representation, as assessed by modulation depth, was not correlated with neuronal firing rate changes.

## Introduction

Nitrous oxide (N_2_O) and ketamine are unique anesthetics with antidepressant (Tadler and Mickey, 2018) and psychedelic (Icaza and Mashour, 2013) effects at subanesthetic concentrations. Unlike canonical general anesthetics, they are thought to (1) act by antagonizing glutamatergic NMDA receptors (Thomson et al., 1985; Jevtović-Todorović et al., 1998) rather than through the potentiation of GABAergic transmission; (2) increase cerebral metabolism (Takeshita et al., 1972; Deutsch and Samra, 1990); (3) enhance high-frequency electroencephalographic activity (Rampil et al., 1998; Lee et al., 2013; Akeju et al., 2016); and (4) increase cortical cholinergic tone (Shichino et al., 1998; Pal et al., 2015). There has been a recent focus on how large-scale brain networks are modulated by general anesthetics in terms of functional connectivity, dynamics, and graph-theoretical variables (Cao et al., 2018; Huang et al., 2018; Lee and Mashour, 2018); however, there is a paucity of mesoscopic network data for ketamine and N_2_O. Analysis of local cortical networks during N_2_O exposure may provide better understanding of the mechanism responsible for the psychedelic, analgesic, and antidepressive properties and may also inform therapeutic strategies.

Neural firing rate, cortical oscillations, and information transfer in nonhuman primate cortex during exposure to anesthetic concentrations of ketamine have previously been characterized (Schroeder et al., 2016). This study investigates the effects of N_2_O on neuronal spiking rate and high-frequency content of local field potentials (LFPs) in primary motor cortex (M1) of the nonhuman primate brain. Furthermore, previous animal studies suggest that somatosensory afferents from somatosensory cortex (S1) mediate transfer of information to M1, where sensory content is represented (Andersson, 1995; Farkas et al., 1999; Mao et al., 2011). Thus, to evaluate the effect of N_2_O on sensory representation in M1, we measured the somatosensory content in M1 during a finger-brushing task. Somatosensory content in M1 can serve as a surrogate of cortical information transfer during exposure to NMDA antagonists such as N_2_O, because content in S1 has been shown to be preserved (reflecting intact thalamocortical information transfer) during anesthetic doses of ketamine while content in M1 was disrupted (reflecting impaired corticocortical information transfer; Schroeder et al., 2016).

## Materials and Methods

### Surgical procedure

**Figure 1.**
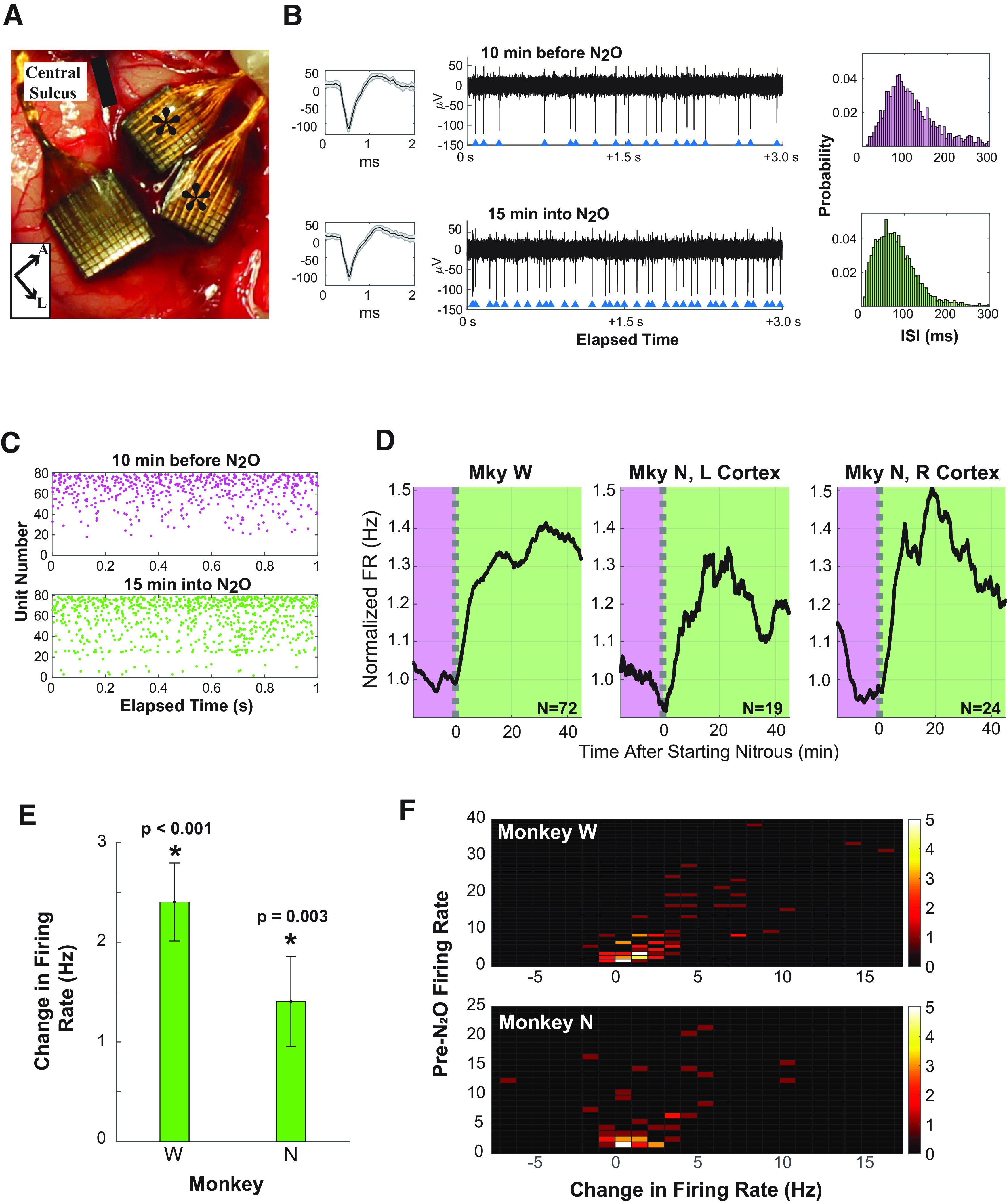
The influence of nitrous oxide on spiking rate. ***A***, Image of the implanted arrays in primary motor cortex. Central sulcus is indicated with a solid black line, and asterisks denote the split motor arrays used in this analysis. The sensory array was not analyzed. The legend at the bottom indicates the anterior direction (A) and the lateral direction (L). ***B***, The single-unit insets on the left show the mean (in black) and SD (in gray) for spike waveforms from 15 to 5 min before N_2_O initiation (top) and from 10 to 20 min after N_2_O initiation (bottom), and indicate waveform stability before and during N_2_O. The *y*-axis of the inset is reported in microvolts. In the middle panel, the raw data from channel 25 shows discriminated single units at 10 min before N_2_O administration (top) and 15 min after beginning continuous N_2_O administration (bottom). The raw data depict increased spiking rate for this channel. The blue triangles indicate the time of an action potential. On the right, spike-interval histograms show the distribution of the interspike interval of the period 10–20 min before N_2_O (top, magenta) and 10–20 min after N_2_O (bottom, green). The area of histograms sums to unity. ***C***, The 1 s raster of Monkey W plot for 1 min before (pre-N_2_O, purple) and 15 min after (green) the start of N_2_O administration is given to illustrate representative results. ***D***, The trend of spiking rate is depicted for each monkey for pre-N_2_O state (purple region) and continuous N_2_O administration (green region). The spiking rate is normalized by the baseline firing rate before N_2_O administration. The pre-N_2_O baseline firing rate was 8.1 Hz for Monkey W, 4.7 Hz for the left cortex of Monkey N, and 6.4 Hz for the right cortex of Monkey N. The number of sorted multiunits for each day, *N*, is indicated with each subplot. The dashed lines indicate when N_2_O was initiated. ***E***, The bar plot depicts the change in spiking rate when combining both days for Monkey W and Monkey N. There is a statistically significant increase in spiking rate for both monkeys, as indicated by the asterisk (*p *<* *0.001 for Monkey W and *p *=* *0.003 for Monkey N). ***F***, The two-dimensional histograms illustrate the number of multiunits with the change in firing rate during N_2_O (on the *x*-axis) and the pre-N_2_O firing rate (on the *y*-axis). The actual number of multiunits is color coded with the legend to the right of the panel. For both Monkey W (top) and Monkey N (bottom), the histogram is shifted to the right.

Experimental protocols were approved by the authors’ Institutional Animal Care and Use Committee. Two male rhesus macaques, Monkey W and Monkey N, were implanted with Utah Arrays with electrode lengths of 1.5 mm (Blackrock Microsystems) in M1 using methods previously described (Schroeder et al., 2016). Monkey W was implanted with two 96-channel Utah Arrays, with one in M1 and one in S1. Monkey N was implanted in left M1 with two 8 × 8 electrode split arrays (where the anterior array was used in this analysis) and one 96-channel array in S1. Because of exposure, the wire bundle connected to the posterior array of Monkey N was cut, which eliminated the signal from 64 of the channels. Because of this damage, Monkey N was eventually reimplanted in the contralateral (right) cortex with two split 8 × 8 electrode arrays in M1 and one 96-electrode array in S1, shown in Figure 1*A*. There were no clinically significant adverse events in addition to wound revision surgeries for exposed hardware from a receding wound edge during healing.

### Experimental setup and N_2_O administration

Three tests were performed on 3 d, separated by several months, for each nonhuman primate: one for Monkey W, one for the left implant of Monkey N (day 1 of Monkey N), and one for the right implant of Monkey N (day 2 of Monkey N). The macaques were trained to sit in a monkey chair [Crist Instrument (http://www.cristinstrument.com)], with their head secured in customized titanium posts (Crist Instrument), while their Utah Arrays were connected to a neural signal processor (Cerebus Neural Processing System, Blackrock Microsystems) and their arms were secured in acrylic restraints. The monkeys were also trained to tolerate finger brushings without agitation. Using a cotton-tip applicator, individual fingers were manually brushed without skin indentation at a 2 Hz rate timed to a metronome. The fingers brushed during a given trial were randomly selected by a computer running xPC Target (MathWorks) and displayed on a monitor to prompt the experimenters. Brushings were conducted for 5 s, and the first 2 s were discarded from the analysis given that the experimenters switched fingers during the first 2 s of each trial. Given the desire for only sensory information in motor cortex, trials in which the monkey moved spontaneously were noted and later discarded from the analysis. See Schroeder et al. (2016) for further details and illustrations regarding the experimental setup.

A semiclosed Mapleson A breathing circuit delivered 70% N_2_O at a continuous rate via sealed face mask secured snuggly around the head with an elastic band. Monkey W was gradually acclimated to the mask over several sessions with rewards. Monkey N tolerated the mask on the first day of training. The face mask was placed 10–20 min before N_2_O administration to allow the monkey to become comfortable with the face mask system. On day 1 for Monkey W before N_2_O administration, the classification of finger brushings was compared for room air and 100% oxygen via sealed face mask, and no significant differences in correct classification of finger brushings were found. This test was not performed on day 2 for Monkey N.

During N_2_O delivery, the monkeys were monitored under direct observation by a laboratory technician, trained graduate student, or physician. During N_2_O administration, monkeys remained awake and cooperative; heart rate and respiratory rate were checked every 15 min. During the final day with Monkey N, only the respiratory rate was monitored to avoid physical contact with the primate, which led to uncooperative behavior. Respiratory rate remained adequate (always >32 breaths/min) throughout the experiments. After N_2_O administration, 100% O_2_ was administered for 5 min, and monitoring was continued for an additional 15 min after the cessation of supplemental oxygen. The monkeys remained awake and did not lose consciousness, as evidenced by widely open eyes and a normal frequency of motor movements and conjugate eye movements. There were no adverse events related to N_2_O administration.

### Front-end processing—analog to cleaned multiunits

The output from the Utah Array was processed in two distinct formats. The first data stream was the raw signal sampled at 30 kHz. This broadband data stream was used for the spectral analysis described below. The second data stream was the time stamp of all recorded spikes for all channels. Spikes were defined using the Cerebus neural signal processor (Blackrock Microsystems) when the 250 Hz high-pass-filtered signal crossed a threshold of −4.5 times the root mean square voltage. The Cerebus system then communicated the detection of a spike to a computer running customized software in the xPC Target environment (MathWorks). The xPC Target computer logged the timestamp of the received spike in 1 ms time bins. This structure allowed the replay of experimental spikes offline as well as trial-by-trial organization that will be discussed in subsequent sections.

In some analyses described below, sorted units were required. Spikes were sorted using Offline Sorter (Plexon). Sorted clusters were scored with a number between 1 and 4. The principal components of threshold-crossing events were calculated and displayed in a two-dimensional space. Sorted units with a score of 4 correspond to when the principal components of the cluster do not overlap with other threshold-crossing events; a score of 3, to clusters with little overlap with other clusters; a score of 2, nonclusters and a morphologic bipolar spike in the time domain; and a score of 1, the remaining threshold-crossing events. Sorted units with a score of 1 were excluded, those with a score of 2 were considered sorted multiunits, and those with scores of 3 and 4 were considered isolated single units.

Because we wanted to include neural information from the hash unit, all threshold-crossing events were used for population-based decoding analysis and for calculating the modulation depth. These unsorted multiunits were reviewed by eye, and channel waveforms that were not consistent with a neuronal action potential were removed from the analysis.

Removing data at times of motor movement was a multistep process. First, during finger-brushing trials, times of movement were flagged by experimenters, and these data were not included in the subsequent analysis. Second, using an automated program, any finger-brushing trial was removed if ≥30 channels recorded a spike in the same 1 ms bin, as this high level of activity was not consistent with a motionless monkey. Third, the raw spike tracings during recordings of Monkey W were reviewed to ensure that no times of high activity (corresponding to movement) were missed. Since there were not additional times flagged, this step was bypassed in Monkey N.

### Data analysis—spike time dynamics

To compare the spiking rate before and during N_2_O administration, the neural data for Monkey N were combined for both days (but the neural data of each monkey were kept separate). The pre-N_2_O data included the 15 min period before N_2_O administration, as this corresponded to the times the monkey was secured in the testing chair without significant motor movements. Pre-N_2_O multiunits with a spiking rate >2 Hz were included, and the spikes in the remaining channels were sorted. The pre-N_2_O spiking rate was then determined by averaging across all sorted multiunits. To include only spontaneous activity, only sorted units with firing rate >0.5 Hz were analyzed. To compute the spiking rate during N_2_O, we averaged the multiunits during the first 45 min of N_2_O (using the same multiunits used in the pre-N_2_O time period). A period of 45 min was chosen to keep the time under N_2_O the same for each day when computing the spiking rate.

Since times of observed monkey movements were not recorded when finger brushings were not being performed, an automated algorithm was used to flag dense neural activity that likely corresponded to monkey movement. When the number of recorded spikes within a 3 ms time bin exceeded a prespecified threshold, the surrounding 1 s interval was excluded from the spike rate calculation. This threshold was empirically determined by reviewing raster plots by visual inspection to ensure that these periods of dense neural activity were excluded. The distribution of sorted multiunits was plotted as a two-dimensional histogram of firing rate change during N_2_O and pre-N_2_O firing rates. To compute the trends in spiking rate versus time, the spiking rate was calculated as a running average over 5 min.

### Spectral analysis

Spectrograms were generated from the broadband signal, sampled at 30 kHz, from the Cerebus neural signal processor using MATLAB (version 2017a, MathWorks). Because removing data from the time series introduces discontinuities in the spectrogram, no time series data were removed (e.g., periods with movement are included in the spectrogram). The signal was first decimated using *decimate.m*, which included a 1000-order FIR (finite impulse response) low-pass filter. After decimation, the signal was high-pass filtered with a 1000-order FIR filter using *fir1.m*. The spectrogram for each channel was approximated using the mtspecgramc.m function in the Chronux toolbox (version 2.12 v03, Chronux), which uses Thomson’s multitaper method, with a time–bandwidth product of 120, a time window of 30 s, 239 tapers, and time step of 15 s. The spectrogram was then averaged across all channels with a spiking rate >2 Hz, as given in the preceding section. The amplitude was normalized such that the maximum amplitude was at 0 dB.

After observing changes in the spectrogram during N_2_O administration, a period of maximal effect between 10 and 20 min after beginning N_2_O was compared with the period 5–15 min before N_2_O. The spectra were again calculated using Thomson’s multitaper method for each channel using the mtspectrumc.m algorithm from the Chronux toolbox using a time–bandwidth product of 120, 30 s windows, and 239 tapers. The data were then averaged across all non-noise channels with a spiking rate >2 Hz in each respective time session. For display clarity, the spectrum for each monkey was normalized by calculating the magnitude at 10 Hz in the pre-N_2_O time period and dividing the entire spectrum in the pre-N_2_O and N_2_O periods by this value.

### Finger classification

Finger brushings were performed while N_2_O was administered, and a detailed analysis was performed on 2 experimental days, one for Monkey W and one for Monkey N (right-sided implant). In both monkeys, finger-brushing sessions were conducted for 10 min, with 5–10 min breaks between sessions. In the first few minutes after beginning inhaled N_2_O, Monkey N often reacted with movement, which required many of these finger-brushing trials to be excluded. Although Monkey W did not have this issue, we excluded the initial finger-brushing session in both animals after beginning inhaled N_2_O to be consistent across both animals.

The remaining three brushing sessions for each monkey were labeled as the pre-N_2_O, early, and late sessions. In Monkey W, the early session began 23 min after starting N_2_O, and the late session began at 45 min. In Monkey N, the early session began 22 min after N_2_O, and the late session began at 38 min. Differences in timing in each session were attributed to ∼10 min breaks between sessions for Monkey W and ∼5 min breaks between sessions for Monkey N.

A naive Bayes classifier with leave-one-out cross-validation was used to classify three or four distinct finger brushings, as previously described (Schroeder et al., 2016), using unsorted multiunits. Again, unsorted multiunits (as opposed to sorted multiunits) were used to retain information in the neural hash unit. The multiunits used to classify finger brushings all had spiking rates >2 Hz. Units were not prescreened with ANOVA to include units with potentially small amounts of information.

On both days, four fingers were brushed and the results of classifying all fingers were included in the following analyses. Three fingers for which the effect could be clearly illustrated were selected for visualization. In both monkeys, fingers 2, 3, and 5 were selected as this combination of fingers in both monkeys had high numbers of modulated units. Because testing periods required motionless and cooperative behavior from the monkey, no attempt was made to use the exact number of trials each day.

In a preliminary analysis using a naive Bayes classifier, sensory content in S1 appeared to be largely preserved with N_2_O during the finger-brushing session with Monkey N, similar to that found in the study by Schroeder et al. (2016). Further analysis of sensory afferents to M1 is warranted.

### Tuning curves and normalized modulation depth

The modulation depth for each brushing session was calculated. The modulation depth between digits *i* and *j* is given below in Equation 1, where μ*_i_* denotes the mean firing rate when digit *i* is brushed and σ*_i_* denotes the SD. The *j*th digit is the finger that minimizes MD*_i_* in Equation 1, since the *i*th digit needs to be differentiated from the three other brushed fingers. The total modulation depth for the channel (MD) was defined herein as the average MD*_i_* for all four fingers as shown in Equation 2. The normalized MD (MD*_N_*) is given in Equation 3, where MD_rand_ is the calculated modulation depth when the fingers associated with the brushing trials are randomly permutated and averaged over 1000 trials. Normalization was necessary because the spiking rates differed across finger-brushing trials and because a finger-brushing session with fewer trials would be biased toward higher modulation depths (for the same reason that the SEM decreases with an increasing number of samples), as follows:

(1) $${\text{MD}}_{i,j}=\frac{\left|\mu_{i}-\mu_{j}\right|}{\sqrt{\sigma_{i}{}^{2}+\sigma_{j}{}^{2}}},$$

(2) $$\text{MD}=\frac{1}{n}\overset{4}{\underset{i=1}{\sum }}{\text{MD}}_{i},$$

(3) $${\text{MD}}_{N}=\frac{{\text{MD}}_{i,j}}{{\text{MD}}_{\text{rand}}},$$

Tuning curves were calculated for all discriminated single units (scores of 3 or 4). The mean and SEM for the spiking rate versus finger brushed are calculated for each of these multiunits.

### Experimental design and statistical analysis

The experimental design, including animal descriptions, is detailed in the preceding sections. Statistical analyses were performed on a desktop computer using MATLAB. Unless otherwise described, statistical significance was deemed as α < 0.05. A one-tailed binomial test was used to evaluate whether finger classification sessions during N_2_O administration performed at a better than chance rate. The chance rates were one in three when brushing three equally likely fingers and one in four when brushing four equally likely fingers. The calculation was made using *binocdf.m* in MATLAB. When two finger combinations were examined for statistical significance (see Population analysis below), a Bonferroni correction was applied to lower the level for statistical significance to 0.025. Otherwise, the level of statistical significance was α = 0.05. Fisher’s exact test was used to compare categorical random variables. Parameterized statistical tests between two groups were made with a one-sample, two-tailed *t* test.

The correlation between MD*_N_* and the N_2_O-induced change in firing rate was evaluated for multiunits during the early and late finger-brushing sessions. The change in firing rate was calculated by subtracting the pre-N_2_O firing rate from the firing rate of either the early or late session. The difference was normalized by the pre-N_2_O firing rate to prevent units with high firing rate from dominating the correlation. The MATLAB function *fitlm.m* was to evaluate the correlation.

## Results

### Increased spiking rate with administration of N_2_O

In motor cortex, the spiking rate increased after exposure to N_2_O. After excluding all channels with a spiking rate of <2 Hz and then spike sorting, Monkey W had 72 sorted multiunits, the left implant of Monkey N had 19 sorted multiunits, and the right implant of Monkey N had 24 sorted multiunits. The raw tracings from channel 25 in Monkey W in Figure 1*B* illustrate the increased spiking rate of a discriminated single unit 15 min into N_2_O administration compared with before N_2_O administration. Included with the raw tracings are the corresponding single-unit waveform and histograms of the interspike intervals before and after N_2_O administration. The raster plots in Figure 1*C* also illustrate the typical increased spiking rate of the total population after 15 min of N_2_O administration.

Previous studies suggest that the maximal effect of N_2_O occurs within 15–20 min of onset (Stevens et al., 1983; Schröter et al., 2012), and Figure 1*D* shows that the change in baseline spiking rate in motor cortex follows this previously reported trend. There is a rapid increase in spiking rate within the first 15–20 min of N_2_O administration. In all cases, the firing rate either peaks or plateaus at ∼20–30 min. The pre-N_2_O baseline firing rate was 8.1 Hz for Monkey W, 4.7 Hz for the left cortex of Monkey N, and 6.4 Hz for the right cortex of Monkey N. The average sorted multiunit spiking rate was increased from 8.1 ± 0.99 to 10.6 ± 1.3 Hz in Monkey W (*p *<* *0.001; two-tailed *t* test) and increased from 5.6 ± 0.87 to 7.0 ± 1.1 Hz in Monkey N (*p *=* *0.003; two-tailed *t* test). The change in firing rate is shown in Figure 1*E*.

Although firing rate generally increased, with a spiking rate increase in 80% of the units analyzed, the effect was not uniform. Figure 1*F* shows a two-dimensional histogram of the change in the firing rate of a multiunit and the pre-N_2_O baseline firing rate of a multiunit. As can be seen in Figure 1*F*, the histogram is centered right of zero, indicating that, as a group, multiunits showed increased firing rate. In some multiunits, there was a substantial increase in firing rate (>10 Hz), while the majority of multiunits increased by only a few hertz. Of note, the firing rate decreased in only 11 multiunits (15%) in Monkey W and 12 multiunits (28%) in Monkey N with N_2_O. There were no observed differences in the waveform morphology in isolated units with increased versus decreased firing rate.

### N_2_O modulation of beta- and gamma-band frequencies

**Figure 2.**
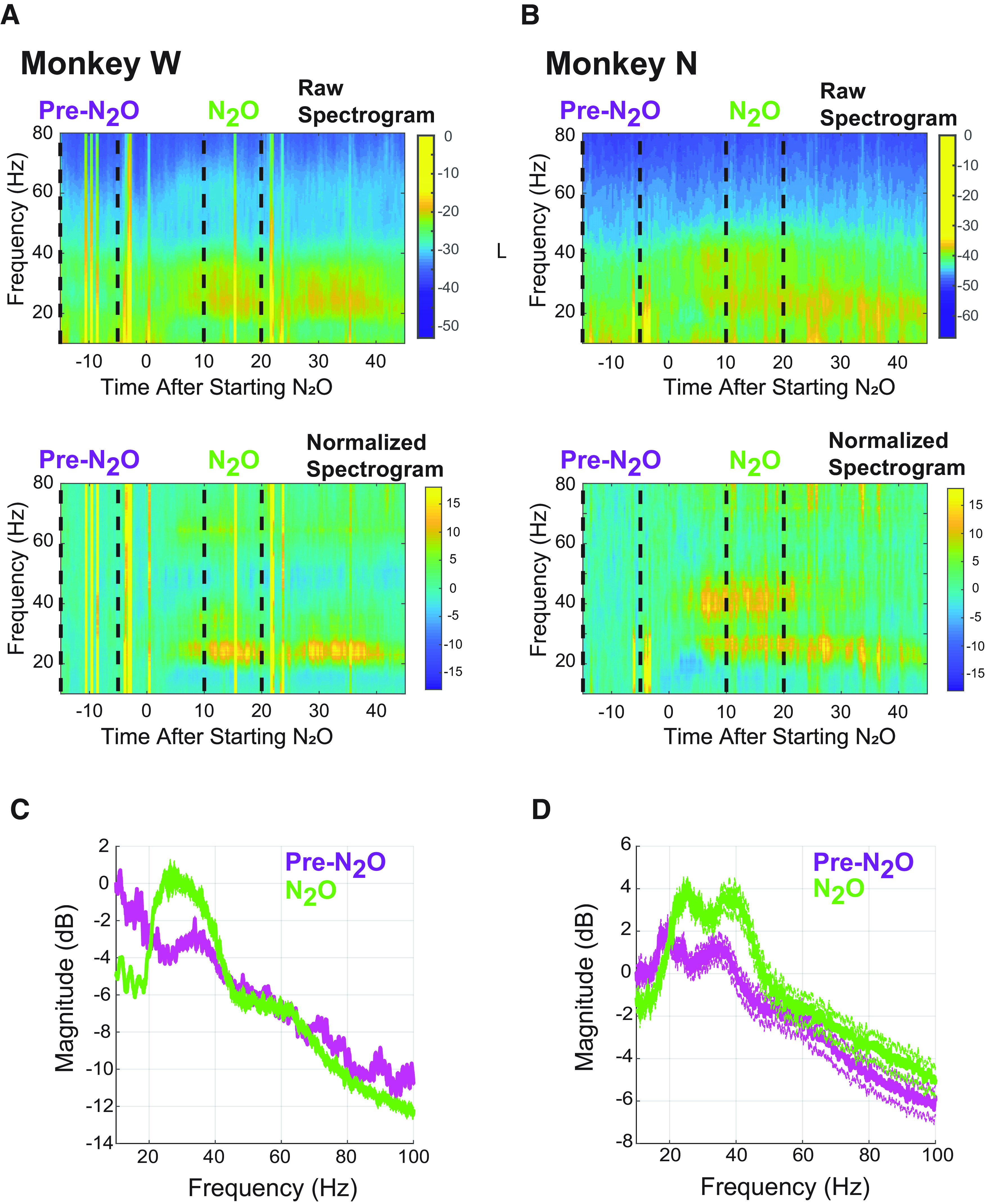
Increased high-frequency power during nitrous oxide. ***A***, ***B***, Averaged spectrograms for 55 channels in Monkey W (***A***) and for 21 channels of the right cortex implant in Monkey N (***B***). The raw spectrograms for each monkey are presented in the top panels of ***A*** and ***B***. The bottom panels depict spectrograms normalized by the average spectral content in the pre-N_2_O period. The vertical lines represent an artifact. ***C***, ***D***, The corresponding averaged power spectrum from 15 to 5 min before N_2_O (purple) and 10–20 min into N_2_O administration for Monkey W (***C***) and Monkey N (***D***). SEM is displayed with dashed lines. To remove the effects of pink noise, the power spectra in ***C*** and ***D*** are normalized by 1/*f*.

In both monkeys, N_2_O administration increased spectral power at frequencies within 20–45 Hz that included both beta band (15–30 Hz) and low gamma band (30–70 Hz). Spectrograms for Monkey W and Monkey N (right implant) are shown in Figure 2, *A* and *B*, respectively, to illustrate the time evolution of spectral changes with N_2_O. Power in the beta and low-gamma band increases beginning at ∼5–10 min in both animals. To highlight the spectral changes under N_2_O, spectrograms normalized based on pre-N_2_O values are shown in the bottom panels of Figure 2, *A* and *B*. Figure 2, *C* and *D*, depicts, respectively, the power spectra for Monkey W and the right implant of Monkey N chosen at the time of maximal effect from the spectrogram, 10–20 min after beginning administration. The comparison of the power spectra before and during N_2_O administration also highlights the increase in spectral power between 20 and 45 Hz. Although times with movement are not excluded from the spectrogram and spectrum, the results are consistent with the results of the power spectra when instances of motor movement are excluded.

### N_2_O degrades but does not eliminate classification of finger brushings

#### Population analysis

**Figure 3.**
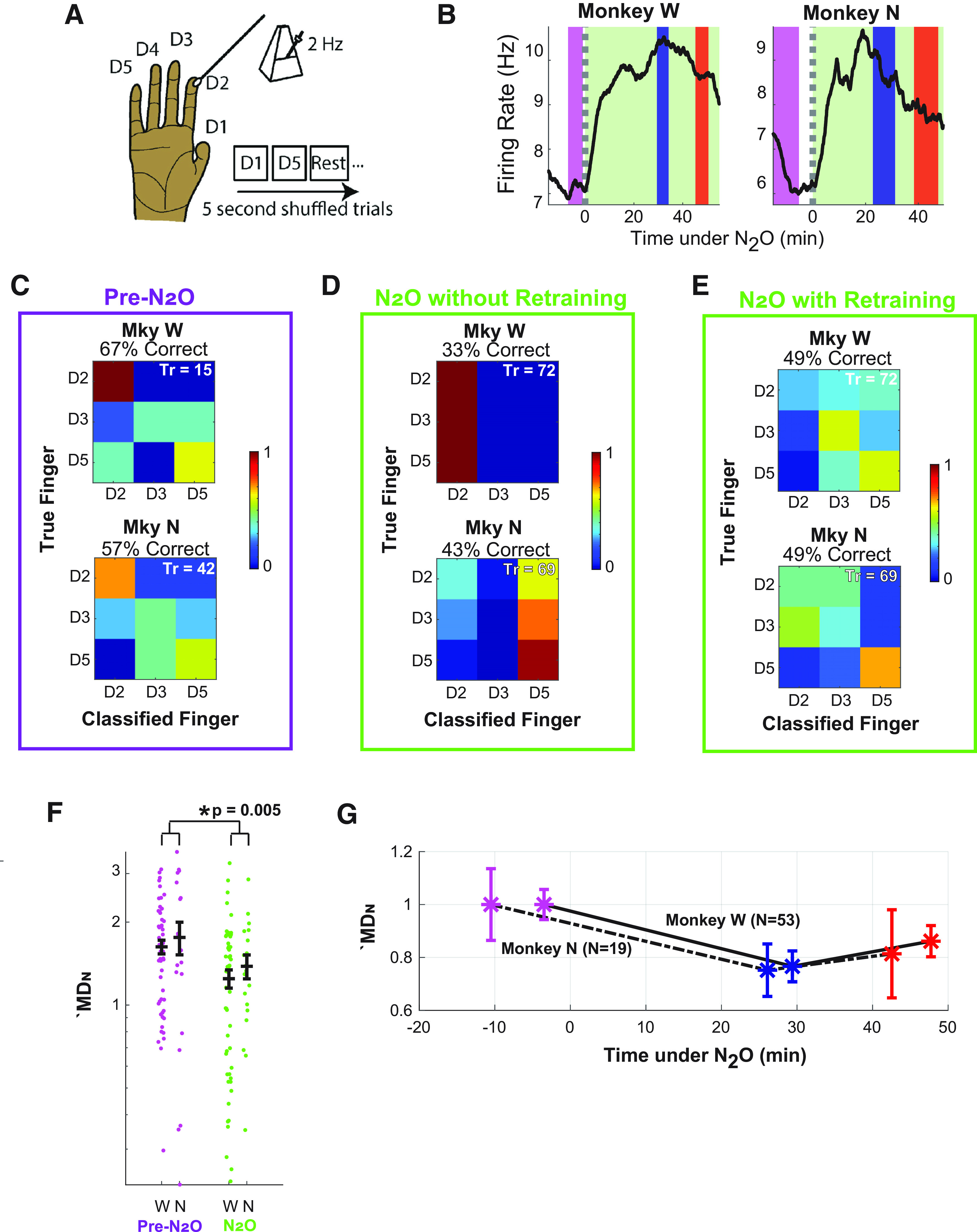
Transient degradation of somatosensory information in motor cortex. ***A***, The experimental setup consisted of random stimulation of individual fingers at 2 Hz strokes in trials of 5 s duration. ***B***, Trends of sorted multiunit firing rate for each monkey. The shaded regions highlight the times when fingers were decoded to compare performance in pre-N_2_O state (purple) and early (blue) and late (red) N_2_O administration. ***C***, Confusion plots for each monkey illustrate performance of the Naive Bayes classifier when classifying finger brushings in the pre-N_2_O state. The vertical axis is the true finger brushed while the horizontal axis is the decoded finger. The percentage correct is given above each respective plot. Tr, Number of trials classified. The insert to the right of the confusion plots is the legend. ***D***, Confusion plot during N_2_O (combined early and late brushing sessions) when the classifier is trained using data from the pre-N_2_O finger-brushing session. ***E***, Confusion plot during N_2_O (combined early and late sessions) when training the classifier on the current session (early and late) using leave-one-out cross-validation. ***F***, The MD*_N_* for all multiunits in the pre-N_2_O (purple) and N_2_O (green) brushing sessions. The MD*_N_* of multiunits during N_2_O sessions was averaged for both the early and late sessions. W denotes Monkey W, and N denotes Monkey N. Each filled circle represents one multiunit. The horizontal bar (in black) and error bars represent the mean and SEM. The asterisk (*) denotes statistical significance. The *y*-axis is in log scale to better visualize the data. ***G***, The mean modulation depths for the pre-N_2_O (purple), early N_2_O (blue), and late N_2_O (red) finger-brushing sessions. The asterisks and error bars indicate the mean and SEM. The solid line denotes Monkey W, and the dashed line denotes Monkey N. Panel ***A*** is adapted and reprinted with permission from Schroeder et al. (2016).

To evaluate information processing associated with spiking and spectral changes, we evaluated the ability to decode sensory information in motor cortex, which is known to represent somatosensory information transferred from S1 (Schroeder et al., 2016). Finger brushings were classified using M1 neurons (Fig. 3*A*). As shown in Figure 3*B*, these somatosensory stimuli were classified at the following three times: pre-N_2_O (purple), early during N_2_O (blue), and late (red). To prevent introduction of bias, classification was performed on all unsorted multiunits with a firing rate of >2 Hz in all finger-brushing sessions. This selection process left 53 units in Monkey W and 19 units in Monkey N. Across trials in Monkey W, there were statistically significant differences in spiking rate, as follows: pre-N_2_O, at 13.41 ± 0.27 Hz, and N_2_O at 19.20 ± 0.27 (*p *<* *0.001; *t* test). Across all trials in Monkey N, there were also statistically significant differences: pre-N_2_O at 8.82 ± 0.19 Hz, and N_2_O at 11.35 ± 0.19 (*p *<* *0.001; *t* test).

In both monkeys and before N_2_O, brushings of four fingers could be classified better than chance (chance level at 0.25). In Monkey W, the proportion of correct classification was 0.50 ± 0.11 (*p *=* *0.014; binomial test, 20 trials). In Monkey N, brushings of four fingers were correctly classified at 0.43 ± 0.067 (*p *=* *0.003; 56 trials). To increase the effect for visualization, the confusion plots shown in Figure 3*C* illustrate the distribution of correct classification using the same three fingers, D2, D3, and D5. For fingers D2, D3, and D5, there were 13 modulated units in Monkey W and 8 in Monkey N (screened with ANOVA test using α = 0.05).

To determine whether N_2_O impacted the encoding of somatosensory information in M1, the Naive Bayes classifier trained in the pre-N_2_O session was used to classify finger brushings during N_2_O administration. For the illustrative fingers (D2, D3, D5), the proportion of correct finger classifications when combining early and late drops were 0.33 ± 0.06 (*p *=* *0.54) in Monkey W and 0.43 ± 0.06 in Monkey N (*p *=* *0.05), as illustrated in Figure 3*D*. However, the correct classification improves when the Naive Bayes classifier is trained using trials from the current brushing session under N_2_O (using leave-one-out cross-validation). For fingers D2, D3, and D5, the percentage correct improved to 0.49 ± 0.06% for both Monkey W (*p *=* *0.005) and Monkey N (*p *=* *0.004), and the confusion matrices are shown in Figure 3*E*. For reference, the correct classification using four fingers was 0.38 ± 0.05 for Monkey W (*p *=* *0.005) and 0.30 ± 0.05 for Monkey N (*p *=* *0.10). Combining trials between both monkeys, there was a statistically significant drop in the correct finger classifications from 0.50 ± 0.06 before N_2_O to 0.34 ± 0.03 during N_2_O (*p *=* *0.018). Thus, N_2_O administration degrades but does not eliminate the representation of somatosensory content in M1.

#### Multiunit modulation depth analysis

Although the encoding of somatosensory information is clearly affected by N_2_O, the effect on individual multiunits requires separate analysis. The MD*_N_* of all multiunits is shown in Figure 3*F*, where MD*_N_* < 1 corresponds to no modulation. In both monkeys, the mean MD*_N_* is greater before than during N_2_O administration (*p *=* *0.005). MD*_N_* decreases from 1.63 ± 0.09 to 1.40 ± 0.10 in Monkey W and from 1.76 ± 0.24 to 1.38 ± 0.17 in Monkey N. Despite this drop, combining the number of modulated channels (MD*_N_* > 2) across both channels revealed an average of 11 modulated units per N_2_O brushing session. However, this is less than the 22 modulated units across both monkeys during the pre-N_2_O brushing session (*p *=* *0.046). When the well modulated units (MD*_N_* > 2) are removed, the classification of D2, D3, and D5 during N_2_O (Fig. 3*E*) drops to 0.43 ± 0.06 in Monkey W (*p *=* *0.054) and to 0.26 ± 0.05 in Monkey N (*p *=* *0.92), and neither was statistically better than chance.

During N_2_O administration, there was no correlation between the modulation depth and the change in firing rate during N_2_O (*R*^2^ < 0.001, *p *=* *0.93), meaning that units with large increases in firing rate were not more likely to have a low MD*_N_*. As will be illustrated with discriminated single units in the next section, some units lost modulation despite no change in spiking rate, and some units maintained modulation despite increased firing rate.

When the early and late N_2_O finger-brushing sessions are separated (Fig. 3*G*), MD*_N_* does not continue to decrease with continued N_2_O administration, despite continued administration of N_2_O. The modulation depth, in fact, increases, although the increase is not statistically significant (*p *=* *0.28).

#### Discriminated single-unit examples

**Figure 4.**
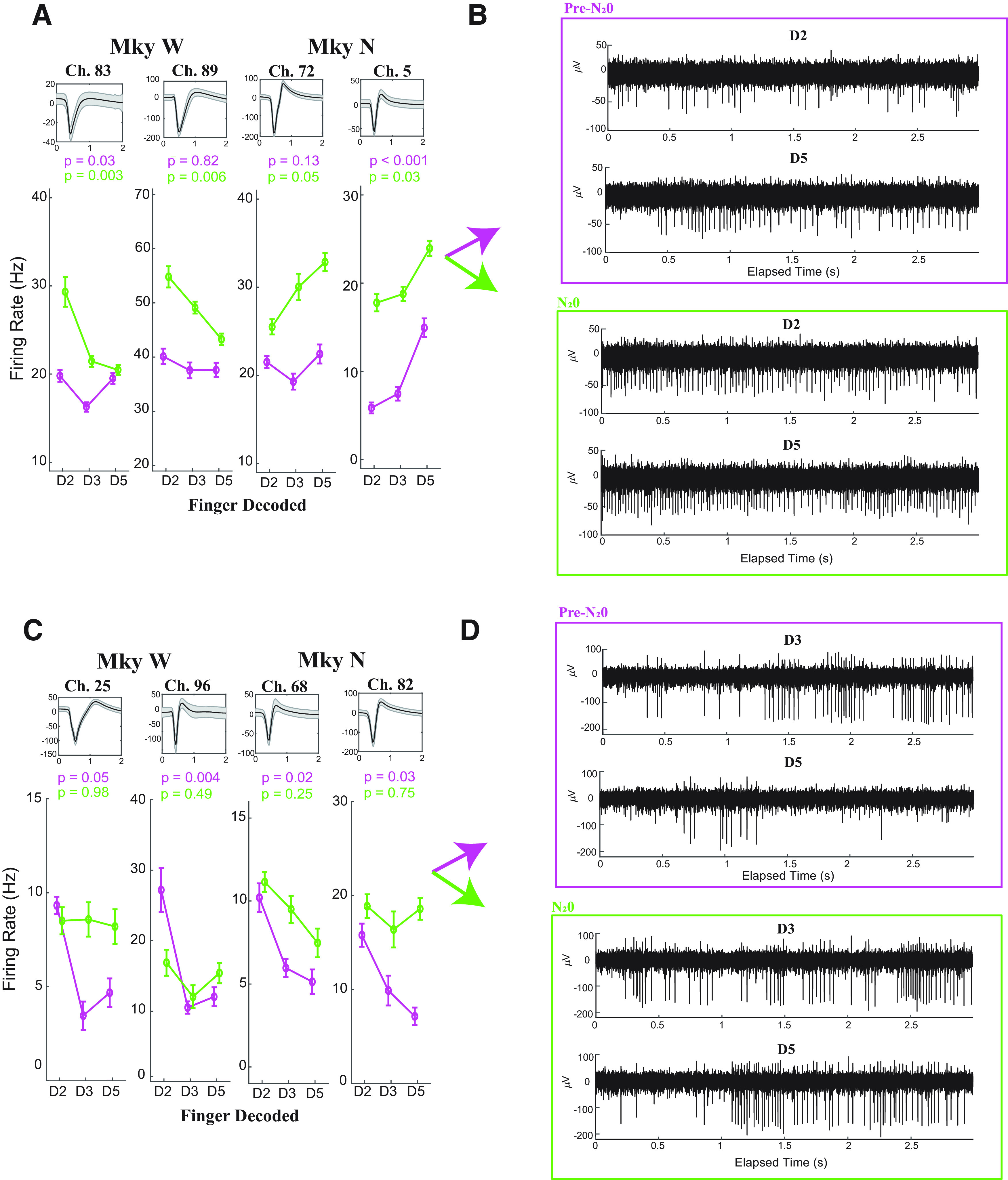
Discriminated single-unit tuning curves. ***A***, Tuning curves for four modulated (ANOVA, with α < 0.1), discriminated single units during N_2_O administration. Single units are presented on the left for Monkey W and on the right for Monkey N. Each single unit is labeled with the channel on the array, the average waveform tracing (black line, mean; shaded gray, SD), and the tuning curve. The tuning curve depicts the mean firing rate of the single unit as a function of the finger brushed both in the pre-N_2_O period (purple) and during N_2_O (green). The error bars denote the SEM. The *p* values calculated with one-way ANOVA are given for both the pre-N_2_O and N_2_O tuning curves. ***B***, Raw voltage tracings for the modulated discriminated single unit in channel 5 for Monkey N in the pre-N_2_O (purple) and N_2_O (green) periods. In each pane, the top tracing resulted when the second digit (D2) was brushed and the bottom plot resulted when the fifth digit (D5) was brushed. The elapsed time is relative to 10 min before beginning N_2_O. ***C***, Four discriminated single units that were originally modulated (ANOVA, with α < 0.1) in the pre-N_2_O period but lost modulation during N_2_O administration. ***D***, Raw voltage tracings illustrating a typical finger-brushing trial for channel 82 in Monkey N. The elapsed time is relative to 15 min after beginning N_2_O.

To better understand how individual neurons representing somatosensory information are affected by N_2_O, we examined the tuning curves of the discriminated single units for each monkey by comparing the pre-N_2_O with the early and late epochs. Combining both monkeys, 19 discriminated units were modulated in at least one finger-brushing session (ANOVA with α < 0.05). Four illustrative examples of the tuning curves for modulated single units are given in Figure 4*A*. The curves illustrate the mean firing rate when one of the three fingers (D2, D3, or D5) was brushed. As mentioned above, despite the increased firing rate, these units retained their tuning for finger brushings. In the raw voltage tracings in Figure 4*B*, the example for channel 5 in Monkey N illustrates how brushing D5 continues to evoke a higher firing rate than D3 despite an increased baseline firing rate. Additionally, there were examples where fingers not tuned in the pre-N_2_O brushing session became tuned to finger brushings, as in channel 89 in Monkey W.

**Figure 5.**
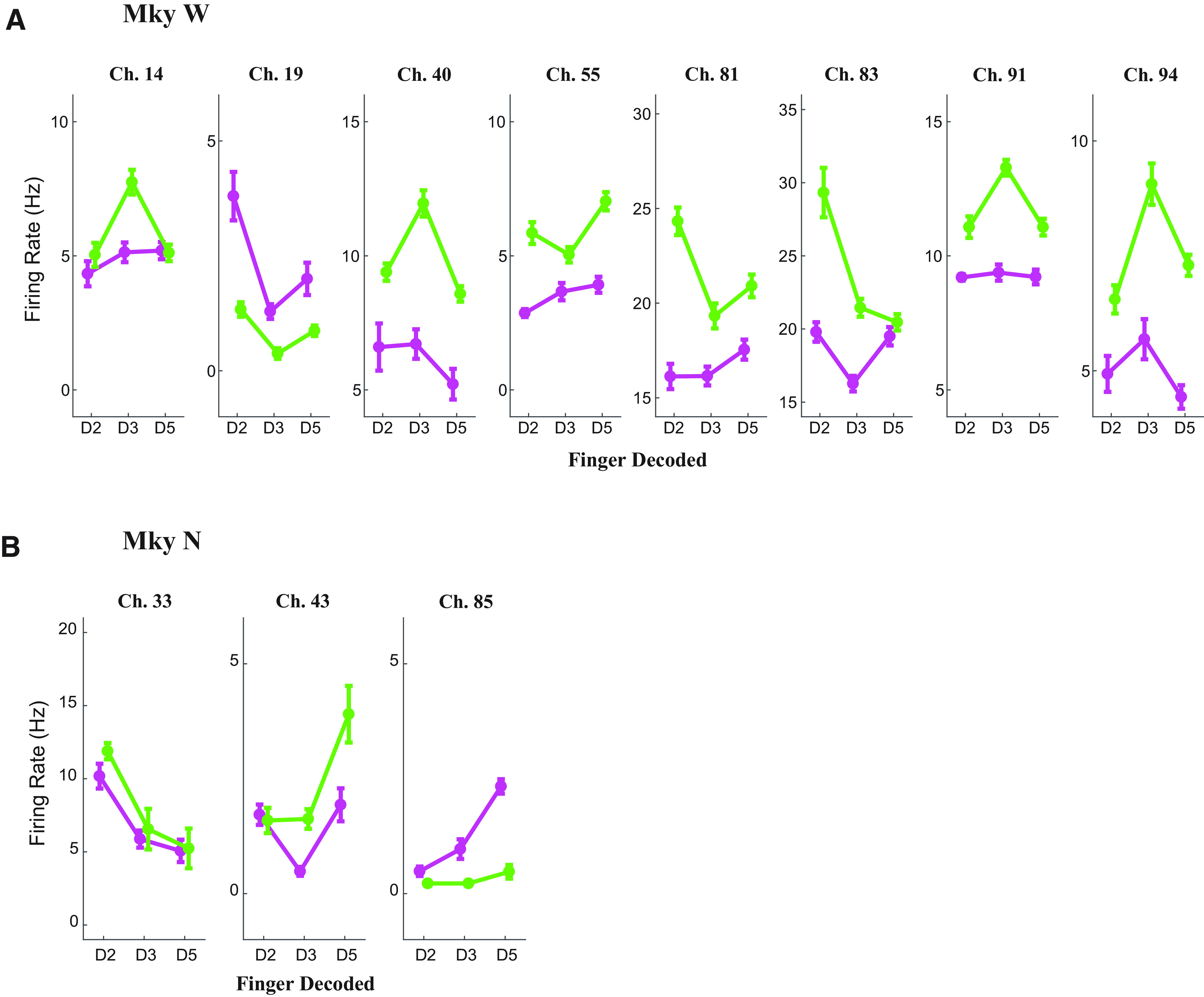
Remaining discriminated single-unit tuning curves. ***A***, ***B***, Monkey W (***A***) and Monkey N (***B***). The tuning curve depicts the mean firing rate of the single unit as a function of the finger brushed both in the pre-N_2_O period (purple) and during N_2_O (green). The error bars denote the SEM.

As described in the preceding section, tuning is lost in many single units during N_2_O administration (Fig. 4*C*). This loss in tuning occurred both in units with increased firing rate (channel 82, Monkey N) and in units with similar firing rates as the pre-N_2_O brushing session (channel 96, Monkey W). Raw tracings of channel 82 in Monkey N illustrate how single-unit activity does not clearly differentiate the brushings of fingers D3 and D5 during N_2_O, although differentiable in the pre-N_2_O session. Thus, regardless of changes in firing rate, many units were no longer modulated during N_2_O. The remaining 11 modulated units are given in Figure 5.

## Discussion

We have demonstrated that, with continuous inhalation of N_2_O, (1) the spiking rate of motor cortex neurons increases, (2) high-frequency power (in beta and low gamma bands) increases, and (3) measurable somatosensory information represented in the motor cortex persists but is degraded while a smaller number of persistently tuned units remain. The change in somatosensory encoding in M1 is explained by a loss of modulated multiunits and a general decrease in modulation across the population. However, there does not appear to be a strong relationship between the increase in single-unit activity and its modulation depth under N_2_O, suggesting a possible dissociation of representation and changes in firing rate.

### Time–frequency dynamics

A paucity of data exists concerning the effects of NMDA receptor antagonists, like N_2_O, on mesoscopic neural networks. Recently, canonical agents that potentiate GABA, such as propofol and isoflurane, have been shown to decrease spiking rate in cortical mesoscopic networks (Lewis et al., 2012; Ishizawa et al., 2016; Wenzel et al., 2019). Wenzel et al. (2019) administered isoflurane at varying concentrations and found a depression of spiking activity in somatosensory and visual cortex that was inversely related to anesthetic depth. Lewis et al. (2012) administered bolus doses of propofol to induce loss of consciousness and observed decreased spiking activity in temporal lobes. Ishizawa et al. (2016) administered propofol at a continuous rate to induce loss of consciousness and also observed decreased spiking activity in somatosensory and frontal ventral premotor cortices with loss of consciousness. NMDA receptor antagonists are well known to variably modulate neuronal firing rate, depending on neuron type and anatomic location (Patel and Chapin, 1990; Homayoun and Moghaddam, 2007; Wang et al., 2013; Schroeder et al., 2016).

Unlike GABA-potentiating medications, N_2_O, an NMDA receptor antagonist, has previously been observed to increase spiking rate in a reticular activating system, but to decrease spiking rate of somatosensory thalamic relay neurons (Kawamoto et al., 1990). In our study, the spiking rate of motor cortex neurons increased with subanesthetic N_2_O. The mechanism for the observed increase in spiking rate is likely because of preferential antagonism of NMDA receptors on inhibitory interneurons that disinhibits pyramidal neurons, as previously shown for the NMDA receptor antagonist dizocilpine maleate (MK801; Homayoun and Moghaddam, 2007). In contrast to N_2_O producing increased spiking rates of M1 neurons, previously reported spiking rates of M1 neurons remained unchanged in nonhuman primates at anesthetic doses of ketamine and an NMDA receptor antagonist (Schroeder et al., 2016). However, at subanesthetic doses of ketamine, Mori et al. (1971) previously showed that multiunit activity increased in the thalamus and reticular activating system of a cat.

Nitrous oxide has spectral properties unlike typical anesthetics potentiating GABA. With microarray recordings, Ishizawa et al. (2016) showed gamma and high beta oscillations lasting a few minutes with propofol-induced loss of consciousness, followed by slow-frequency delta oscillations. In particular, after beginning propofol infusion but before loss of consciousness (i.e., subanesthetic doses), spectral plots suggest an increase in high beta (18–25 Hz) in S1 and in low gamma (25–34 Hz) in ventral premotor cortex that is similar to our spectral findings with subanesthetic N_2_O. Lewis et al. (2012) likewise found the appearance of slow (<1 Hz) oscillations in local field potentials after loss of consciousness induced by propofol. In spectral analyses of anesthetic doses of ketamine, Lee et al. (2013) showed a relative increase in gamma band power and a decrease in beta-band power in frontal/parietal EEG data. Schroeder et al. (2016) demonstrated the same trends in LFPs of S1 and less obvious trends in M1. Likewise, Ballesteros et al. (2020) found a gradual increase in high beta, low gamma power in ventral premotor cortex, S1, and secondary somatosensory cortex. Although there are no mesoscopic LFP spectral data with N_2_O, previous spectral analyses with EEG data show that N_2_O can increase and decrease high-frequency power (Yamamura et al., 1981; Rampil et al., 1998; Foster and Liley, 2011, 2013; Pelentritou et al., 2019). We found increased high-frequency (>20–45 Hz) power in motor cortex within beta and gamma bands. Within the beta band, the increase was seen primarily in high beta activity (∼25 Hz) as opposed to the low beta band (∼15 Hz). While both high and low beta bands are thought to originate in cortical layer 5, the high beta band is thought to be associated with sensory stimuli leading to sustained movement, while the low beta band is associated with coordination of cell assemblies in the absence of sensory stimuli and may be important for processes such as working memory (Roopun et al., 2006; Kopell et al., 2011; Gelastopoulos et al., 2019). Within the gamma band, the changes are similar to the increased gamma power described by Yamamura et al. (1981), who reported a peak at 34 Hz with 70% N_2_O; and Rampil et al. (1998), who reported dual peaks at 40–50 and 70–110 Hz with 50% N_2_O. The gamma power differences in the literature may be attributed to regional variation or to the lower spatial resolution of EEG leads, as suggested by others (Ray et al., 2008; Eagleman et al., 2018).

### N_2_O degrades M1 somatosensory representation

Anesthetic agents are diverse in terms of their molecular targets and effect on neuronal activity. Despite these differences, they all share a similar functional property of reversibly suppressing consciousness at anesthetic doses or altering cognition at subanesthetic doses. The mechanism by which GABAergic anesthetics disrupt neural function appears to be clear—they potentiate inhibition and reduce neural activity (Boveroux et al., 2010; Schrouff et al., 2011; Hudetz, 2012). However, it is not clear how non-GABAergic anesthetics affect consciousness or cognition. As discussed above, the NMDA receptor antagonists ketamine and N_2_O increase high-frequency oscillations and neural firing rates. While GABAergic drugs depress information transfer, non-GABAergic drugs like ketamine and N_2_O are thought to disinhibit pyramidal neurons, leading to dysregulated activity that disrupts information processing (Homayoun and Moghaddam, 2007). Our data show that there is decreased somatosensory content that is not simply explained by interference from the increases in firing rate, because firing rate changes were not correlated with modulation depth (i.e., neuronal tuning). Furthermore, examples of single units without changes in firing rate were found to lose tuning to finger brushing, and other single units with increased firing rates were also found to remain tuned to finger brushings. Thus, our data in nonhuman primate cortex do not support the hypothesis of a clear causal link between increased neural firing patterns and disruptions in information transfer/representation. Further studies are needed to clarify the exact influence of N_2_O-induced changes in firing rate on somatosensory representation.

### Limitations

One limitation of this study is that only motor cortex was studied. However, motor cortex was chosen to emphasize the effects of corticocortical somatosensory pathways, although other somatosensory pathways exist, including thalamocortical or corticothalamocortical pathways (Petrof et al., 2015; Mo and Sherman, 2019). Great care was taken to avoid skin indentation with finger brushings, and recording periods with motor movements were excluded to reduce the contribution of thalamocortical inputs. Although this data exclusion has the potential to introduce bias, identical methods for excluding movement artifacts were used throughout all finger-brushing sessions. Second, S1-to-M1 transfer was not measured directly. However, the sensory brushings used in this study have been previously validated as a model of S1-to-M1 information transfer using anesthetic doses of another NMDA antagonist, ketamine, where S1 content was largely preserved and M1 content was lost. Although transfer from S1 to M1 was not explicitly studied here, S1 somatosensory preservation was anecdotally confirmed on 1 d with Monkey N using a Naive Bayes analysis. Thus, we feel that M1 somatosensory content is a likely surrogate for transfer from S1 to M1. However, the delineation of a more detailed effect of N_2_O on specific somatosensory afferents requires further study. Additionally, a possible confounder to interpreting the drop in accuracy of finger classification would be if an alternate process (e.g., agitation, fear) interfered with somatosensory content. However, preliminary control tests did not show significant differences in the number of finger brushings correctly classified with or without the face mask. Last, N_2_O was not delivered in a closed circuit accompanied by high-fidelity gas monitoring, creating the potential for undetected alterations in N_2_O, oxygen, or carbon dioxide. However, a tight mask seal was ensured at the beginning of each experiment, and monkeys remained awake and cooperative with normal breathing rate and respiratory excursions. Thus, a significant confound that would mitigate the interpretation of these results is not likely.

### Conclusion

In this study, we investigated the effects of subanesthetic concentrations of N_2_O on mesoscopic networks in primary motor cortex of the nonhuman primate. Both spiking rate and high-frequency spectral content of motor cortex neurons increased in response to N_2_O. With ongoing N_2_O, measurable somatosensory content and number of modulated units in M1 decreased but were not eliminated. The shift to faster dynamics is not clearly associated with the somatosensory representation on a neuronal level and may suggest additional mechanisms of N_2_O that alter perception.

## Synthesis

Reviewing Editor: Karen Davis, Krembil Research Institute and University of Toronto Decisions are customarily a result of the Reviewing Editor and the peer reviewers coming together and discussing their recommendations until a consensus is reached. When revisions are invited, a fact-based synthesis statement explaining their decision and outlining what is needed to prepare a revision will be listed below. The following reviewer(s) agreed to reveal their identity: Andrew Hudson.

The current study investigated the effects of commonly used anesthetic agents, N2O at a sub anesthetic concentrations to characterize the agent’s effect on the cortical oscillations and multi-unit spiking activities using two non-human primates with Utah arrays implanted in both the primary motor cortex and the somatosensory cortex. This interesting study reports the effects of 70% nitrous oxide on encoding of sensory information in M1 of macaque monkeys. The recording of single unit activity in primate motor cortex during administration of nitrous oxide is a significant contribution to our understanding of how this anesthetic impacts the cortical circuit. Primate neurophysiology is a great deal of work and the authors should be commended for the effort involved. The study found the both spiking rate and high-frequency spectral content of motor cortex neurons increased in response to N2O, specifically that, during nitrous oxide administration, (1) firing rates increase , (2) beta and gamma band power increases in the local field potentials, (3) fewer M1 neurons showed somatosensory tuning, and (4) modulation depth of somatosensory responses was decreased. The paper is clear and well-written, but the issues below should be addressed:

Major Comments

1. Methodologically, the circuit used to deliver the nitrous to the animal should be described. Was this a closed circuit system or a Mapleson circuit? Was end-tidal N20 and CO2 monitored during the experiment? Was the temperature of the animal monitored during the experiment? While the respiratory rate remained unchanged, there is no comment on the minute ventilation. Without monitoring the end tidal CO2 it is possible that shallow breaths could lead to acidemia from hypercarbia and resulting sympathetic nervous system activation that could potentially contribute to the changes observed between the early and late administration of nitrous oxide during the experiments.

2. The authors could consider a more theoretically justified approach to their spectrogram calculations, which currently involve overlapping Hamming window estimates that are then smoothed with a 15 sample median filter, suggesting they have smoothed the data in the time domain extensively. A more principled approach would be to utilize a Thomson multitaper estimate with a defined time-frequency product to set the temporal and frequency resolution at a known value. This offers a clear resolution to the reader that is understandable without obscuring potentially significant temporal dynamics. (I suspect that the median filter was an attempt to suppress movement related artifacts, but if they are present within a single window it is possible to visually integrate over them.) It is probably also worth considering displaying the spectrogram as the variance from the baseline spectrum or mean spectrum over the whole experiment to emphasize the ongoing dynamics.

3. The abstract and introduction need to provide a clear motivation as to why this study is important as it is currently a weakness of the study There were many detailed citations and arguments as to why the study and the specific approaches taken could be motivating, so it is strongly encouraged that the authors revise the introduction to motivate the readers. Specifically, why somatosensory content transfer is interesting to look at for this specific agent, N2O. Also although the authors mentioned, "Network-level dynamics may provide insights into the unique properties of these drugs", what is the network here? What is the justifications or reasons why no signals from S1 which appeared to be possible to be simultaneously recoded with M1 activities? The authors kept using the argument for the somatosensory information transfer from S1 to M1, but since it is not clear what kind of changes, even the same measure used in this study for S1 activities, and if the M1 is heavily affected by the activity in S1, then what are we seeing exactly in this study?

4. The authors state on manuscript page l6 "When the early and late N2O finger-brushing sessions are separated (Fig. 3G), MDN does not continue to decrease with continued N2O administration, despite increasing concentration ofN2O in the brain." Given that the brain concentration must be in equilibrium with the alveolar concentration, it is not likely to be meaningfully increasing in concentration at 45 minutes vs 23 minutes. (I would be very surprised if a GASMAN simulation shows otherwise, given that the FA/FI ratio is >0.9 at 10 minutes.)

5. I am also intrigued by the observation that the disruption of sensory encoding is independent of the firing rate change. It is unfortunate that the sensory array data was not analyzed for the purposes of this report, as it would be very interesting to understand if the loss of sensory tuning in S1 was comparable or if the breakdown was in the communication between S1 and M1. (While the authors wisely refrain from speculating about the mechanism for the loss of sensory encoding, I suppose at a minimum the author’s data argues that the impact of nitrous is not limited solely to a local excitatory effect on the recorded neurons, as then the increase in firing rate would correlate with the loss of sensory modulation depth.) On Page 3: The place of (1) in ...", they (1) are thought to act..." should be changed to ", they are thought to (1) act" to maintain the parallel structure.

Other specific comments:

1. Page 4: It seems that based on the Figure 1A, two split arrays are mentioned for both sides of the implants in M1 and it is assumed that those split arrays are in the same configuration. However, the it seems that, based on the figure, there are two 8 x 8 = 64 ch arrays. Isn’t that true? Please clarify. Also to orient the readers, it would be better to have at least medial-lateral orientation indicated in the figure.

2. page 4: The effects that were described in the manuscript are assumed to be more on the systemic responses, as opposed to layer specific. Some of the details can stay in the cited reference, Schroeder Et. Al. 2016, but it would be useful to mention the depth/length of the electrode contact location/shank.

3. page 8: Is it correct that the spike rate was calculated as a running average over 5 min, as opposed to 5 sec? Please confirm.

4. pages 8-9: It would be better to bring a description of the use of Matlab before start showing names of .m functions. Only on page 9, when the use of pwelch.m was introduced, "in MATLAB.." was introduced. When FIR filters were used, what were the filter specifications for both operations?

5. page 9, it is not clear the normalization procedure described here, "... the spectrum was normalized by the value at 10 Hz in pre-N2O time period ...". Please describe exactly what’s been done here and actually normalized?

6. page 13: It would be more informative if Figure 1.F can have change in firing rate vs baseline rate and the number of units are color coded to show how many units are there for each box or dot. Then it can be seen if there are normal distributed cells and some outliers that change the rates significantly more or not and their relation to underline rates.

7. pages 13-14: Although very wide-band arguments can be informative with regards to beta and low gamma increase, it would be more constructive to subtract underline pink (1/f) like distribution from the PSDs and also not simply just increase in beta and gamma band pawer, but also their shift. Also what does the occasional low beta burst especially prominent in Monkey N in Fig 2 B reflect? After each burst, there are some movement onsets so that beta attenuation was observed.

8. page 14: Again, although both monkeys had arrays in both M1 and S1, and the fact that the authors mentioned the sensory information from S1 to M1, why there is no actual transfer from S1 to M1 is not computed or quantified?

9. page 14 and Figure 3: Based on how the experiment was done and how the timings were logged, it is not clear if clean evoked responses can be obtained from the data and show the durations of evoked responses especially in LFPs. It is the first step to show population response due to N2O, since all neural responses characterized in this manuscript increased, population spike rates, beta, and low gamma band power, it is much more informative as to show if they all increase at the same time, or if they have different time courses.

10. page 15: No units were modulated for D5? How many of those 16 and 8 units are responding to only single digit? Were they modulated to all three digits, D1, D2, and D3?

11. page 15: Since there were only 16 and 8 units total from each monkey respectively, it would be informative to have supplementary materials showing modulation changes for each unit (beyond what’s been presented in Figure 4 for unit analysis), especially since the retraining improves the classification performance, this small population change would be indicative of modulation pattern changes induced by N2O.

12. page 15: Although authors claim that "Thus, N2O administration degrades but does not eliminate the transfer and/or representation of somatosensory content in M1", since the performance map in the confusion matrices for both monkeys changed from pre-N2O state to N2O with or without retraining, and no illustrations of the weights for the classifiers used, it seems that the gross information of the transfer did not change (at the level that each finger sensory information remained somewhat intact), but that encoding/representation of the somatosensory content in M1 did change. Please clearly support the claim in the current manuscript or change the writing.

13. page 17: For Figures 4B and 4D, what does time 0 denote here?

14. page 17: "(3) measurable somatosensory information transferred to the motor cortex persists

but is degraded while a smaller number of persistently tuned units remain" , the authors mentioned the information transfer from S1 to M1, but again the work presented here only represents the representation and decoding performance from peripheral to M1 only. The suggestion made at the end of the page 17 to the beginning of page 18 needs to be expanded further and highlight the weakness of the current study.

15. page 20: After listing a series of studies indicating changes in more specific/narrower bands, the authors mentioned about their finding of "high frequency (>20-45Hz). As mentioned above, by adding all the previous studies as well as the current study, it seems that N2O (and other agents used in other studies) are acting upon a whole beta or gamma ranges, especially for LFPs.

16. The authors should include the specific version number of Matlab in addition to the functions that they used for the current study.
